# Model‐Free and Model‐Based Learning in Human Fear Conditioning

**DOI:** 10.1111/psyp.70349

**Published:** 2026-06-30

**Authors:** L. E. Stemerding, A. M. V. Gerlicher, F. L. Reinhold, M. Kindt

**Affiliations:** ^1^ Department of Clinical Psychology University of Amsterdam Amsterdam the Netherlands

## Abstract

Learning to predict threat and to adapt behavior accordingly is critical to human survival. This type of learning is governed by distinct systems in the brain. Model‐free learning is reflexive and computationally efficient, allowing for rapid responses based on past experience, but is also resistant to environmental change. In contrast, model‐based learning is more flexible and relies on an internal model of the environment that can be updated without direct experience. To enhance the translational value of human fear‐conditioning research and facilitate the interpretation of fear‐reduction interventions, we examined whether commonly used measures of conditioned responding reflect model‐free and/or model‐based learning. Based on prior observations and the defensive‐reflex nature of the fear‐potentiated startle (FPS), we hypothesized that FPS responses would reflect model‐free learning, whereas skin conductance responses (SCRs) would reflect model‐based learning. To test this hypothesis, we decoupled the experienced US value (i.e., model‐free) from the expected value (i.e., model‐based) by US devaluation/revaluation manipulations delivered via instructions (Exp1), physical disconnection of the US electrode (Exp2), or their combination (Exp3). By design, instantaneous changes in conditioned responding, prior to any new learning by experience, must indicate model‐based updating rather than model‐free cached value. Across three fear‐conditioning experiments (total *n* = 131), we found that SCRs most likely reflected model‐based learning, whereas FPS responses reflected aspects of both model‐free and model‐based learning. These results show that common indices of conditioned fear are not simply interchangeable: they tap distinct learning systems, and treating them as equivalent risks missing the more affective, experience‐driven components of fear—thereby limiting translation to clinical intervention.

## Impact Statement

To enhance the translational value of human fear‐conditioning research and facilitate the interpretation of fear‐reduction interventions, it is essential to determine what type of learning underlies commonly used measures of conditioned responding. Although it was well established that different physiological measures do not always align, we show here that these measures may reflect distinct underlying learning systems. Our findings can advance critical examinations of the learning mechanisms central to interventions that aim to attenuate maladaptive fear behavior.

## 1 Introduction

Across species, associative learning enables organisms to use past experience to predict potential threats and rewards from environmental cues, supporting rapid defensive action when danger is present. However, adaptive functioning also depends on the ability to revise learned defensive responses when circumstances change—specifically, to attenuate conditioned fear when danger is no longer likely. The inability to update learned behavior under changed circumstances results in the maladaptive and persistent responses that characterize fear and anxiety disorders (Kindt 2014). Contemporary reinforcement‐learning accounts distinguish between a relatively inflexible model‐free system that updates cached value primarily through direct experience, and a more flexible model‐based system that can adjust responding when the environment changes without requiring additional outcome experience (Daw et al. 2005; Doya 1999; Dolan and Dayan 2013). Modeling these distinct forms of learning in experimental settings allows researchers to better understand how maladaptive fear responses can be attenuated. Threat learning and its modification are commonly studied with Pavlovian fear‐conditioning paradigms, in which physiological measures such as skin conductance (SCR) and fear‐potentiated startle (FPS) index conditioned responding (Beckers et al. 2023; Leuchs et al. 2019). Because model‐free and model‐based learning strategies can operate in parallel, different read‐outs of conditioned responding may differentially capture these processes (Kurdi et al. 2019; Pool et al. 2019). Yet it remains unclear which learning processes SCRs and FPS responses reflect when conditioned responding should update as circumstances change, rather than simply persisting because it was learned before. Further insights into the distinction between flexible (model‐free) and inflexible (model‐based) responses in human fear conditioning can improve the interpretation and clinical translation of fear‐conditioning findings.

While Pavlovian conditioning was traditionally considered model‐free (based on the Rescorla‐Wagner learning rule), there is now extensive evidence from both human and animal literature that Pavlovian conditioning can also be model‐based (Dayan and Berridge 2014; Pauli et al. 2019; Prévost et al. 2013; Robinson and Berridge 2013; Talmi et al. 2019). The key difference between model‐free and model‐based learning in Pavlovian conditioning is the extent to which changes to the value of or motivation for the US (i.e., US revaluation) affect responses to the CS (Dayan and Berridge 2014). In model‐free learning, the response to the CS is only updated after direct experience of changes in US value or expectation, while in model‐based learning these changes are instantly reflected in the conditioned response. It is important to note, however, that in classic fear‐conditioning and extinction paradigms, it is not possible to distinguish between model‐free and model‐based learning, because the aversive outcome (or its omission) is typically directly experienced. In humans, the capacity for model‐based learning during Pavlovian conditioning is therefore evidenced by studies showing that the devaluation of the US immediately affects conditioned responses to an associated CS (Hosoba et al. 2001; Schultz et al. 2013). In such studies, US devaluation can either take place through instructions about the intensity or occurrence of the US (e.g., instructed extinction or reversal learning) or through uninstructed changes in the environment or the motivational value of the US (e.g., removing US electrodes or desensitizing the participant to the US). Thus, understanding how various measures of conditioning respond to US devaluation in human fear conditioning could provide insights into whether these measures are more likely to reflect model‐free or model‐based learning.

Previous research indicate that fear potentiated startle responses (FPS) and skin conductance responses (SCR) can show distinct response patterns across manipulations, suggesting that they may reflect partially distinct learning mechanisms (Hamm and Weike 2005; Leuchs et al. 2019; Ojala and Bach 2020). Fear‐conditioning studies that allow for the differentiation of model‐free and model‐based learning suggest that compared to SCRs, FPS responses are governed by more habitual control (Hamm and Weike 2005). For example, instructed reversal of contingencies in a fear‐conditioning paradigm (including instructed extinction) instantly affects SCRs, while both amygdala activation and FPS responses update only after experiencing the new contingencies (Atlas 2019; Javanbakht et al. 2017; Sevenster et al. 2012). Others, however, found that FPS responses too, update immediately following both instructed US presence and instructed US omission (Costa et al. 2015; Mertens and De Houwer 2016). In addition, cognitive reappraisal of the US can reduce SCRs but does not update FPS responses (Dar and Asthana 2026). It should be noted, however, that very few of these studies investigated FPS, and none have directly compared these measures. There is thus robust evidence that SCRs are sensitive to contingency information and the explicit expectation of an outcome, suggesting that SCRs are governed by model‐based learning processes. On the other hand, FPS responses appear to reflect model‐free learning in some instances, but not all. These observed differences could be due to variations in experimental designs, such as initial conditioning strength and certainty of instructions. For example, in the study by Costa et al. (2015) the CS+ was instructed to be followed by a US in the conditioning phase, but never actually reinforced (i.e., participants never experience the US), which could explain the decrease in FPS responses upon instructed reversal. Alternatively, FPS responses may reflect a mixture of model‐free and model‐based learning. This is also suggested by work on instructed extinction, which shows that although FPS responses initially remained elevated following instructions, they declined more rapidly compared to a control group that received no instructions (Sevenster et al. 2012). A more systematic investigation of how different indices of conditioned fear map onto model‐free and model‐based learning systems may yield valuable insights into the specific learning processes contributing to fear‐conditioning and environmental change.

In the current article we present three experiments that investigated the extent to which SCRs and FPS responses during a human fear‐conditioning task are governed by model‐free versus model‐based learning. According to a model‐based framework, environmental information about changes to the US value or occurrence (e.g., verbal instructions or removal of the electrode) should result in immediate updating of conditioned responding, whereas a model‐free framework predicts updated responses only after experiencing the change. All experiments therefore consisted of three phases: a conditioning phase, a US devaluation phase, and a US revaluation phase. Specifically, in the first experiment we investigated the effect of instructed US devaluation, whereas in the second and third experiment US devaluation was operationalized by removing the US electrode. In the third experiment only, the electrode removal was also accompanied by instructions. Given the defensive‐reflex nature of fear‐potentiated startle (FPS; Hamm 2015), and in view of previous observations that SCRs are more responsive to non‐experienced information than FPS responses, we hypothesized that SCRs would update immediately following US devaluation and revaluation, consistent with model‐based learning. In contrast, we hypothesized that FPS responses would update only after direct experience of these changes, consistent with model‐free learning.

## 2 Methods

### 2.1 Participants

Across the three experiments, 132 participants were recruited from the student population of the University of Amsterdam (*n* = 40 in Experiment 1, *n* = 40 in Experiment 2, *n* = 52 in Experiment 3). Exclusion criteria were age < 16 or > 35, suffering from tinnitus, and previous participation in a fear‐conditioning experiment. In Experiment 2, one participant was excluded due to a technical error, resulting in 39 participants being included in the analyses. Demographic characteristics of the participants can be found in Table 1. The experiments were approved by the local ethics committee (Ethical Review Board) of the University of Amsterdam and all participants were informed about the procedures and signed consent before participating. Participants received course credit or financial reimbursement for their participation.

**TABLE 1:** Demographics and participant characteristics.

	Experiment 1	Experiment 2	Experiment 3	*p*
Total *n*	40	39	52	
Gender (m/f)	15/25	9/30	18/34	
Age	21.8 (8.3)	20.7 (2.2)	21.5 (3.0)	0.648
ASI	14.2 (8.4)	15.0 (8.3)	20.2 (9.5)	**0.002**
STAI‐T	38.9 (9.5)	39.6 (9.9)	42.7 (11.5)	0.178
STAI‐S	35.5 (8.6)	34.5 (6.4)	37.4 (11.0)	0.301
US intensity strong (mA)	15.1 (11.4)	24.3 (20.4)	23.3 (16.3)	**0.023**
US rating strong (1–10)	6.8 (1.5)	7.9 (0.6)	7.5 (1.2)	**< 0.001**
US intensity weak (mA)	0.7 (0.7)	—	—	
US rating weak (1–10)	1.3 (0.7)	—	—	

*Note:* The *p*‐values are reported for a one‐way ANOVA comparing scores between experiments. Post hoc tests showed that ASI scores were higher in Exp3 compared to both Exp1 (*p* = 0.004) and Exp2 (*p* = 0.017). US intensity was lower in Exp1 only compared to Exp2 (*p* = 0.037). US ratings were lower in Exp1 compared to both Exp2 (*p* < 0.001) and Exp3 (*p* = 0.007). Bold values indicate statistical significance (*p* < 0.05).

### 2.2 Measures and Materials

#### 2.2.1 Conditioned and Unconditioned Stimuli

Conditioned stimuli were two geometric symbols (triangle or square) that were presented in the centre of the computer screen. Assignment of each symbol to CS+ and CS− was counterbalanced. The unconditioned stimulus was a 2‐ms electrical stimulus applied to the wrist of the left hand through 2 Ag/AgCl electrodes using a constant current stimulator (Digitimer DS7A; Hertfordshire, UK). Before the start of the experiments, the intensity of the electric stimulus was calibrated together with the participants. Starting at 1 mA, the intensity was slowly increased in steps of 2–4 mA. Participants were asked to say stop when the stimulus was clearly uncomfortable and subsequently rated its intensity on a scale from 0 to 10 (0 = “I do not feel anything” and 10 = “the most uncomfortable stimulus I can imagine to be applied via such an electrode”). If participants rated the final stimulus below 7, they were asked to try one intensity level higher, but they were also allowed to proceed without doing so. In Experiment 1, participants were also asked to say stop at the level that was judged as just perceivable, which was used as weak US in the devaluation phase.

#### 2.2.2 Skin Conductance Responses (SCR)

Electrodermal activity was recorded from the middle phalanges of the index and middle finger of the left hand using two AG/AgCl Electrodes of 20 by 16 mm. The signal was recorded using in‐house software (VSRRP) through an input device with an excitation voltage (5 V) of 50 Hz and was digitized at 1000 Hz using a 16‐bit AD‐converter. Subsequently, the signal was analyzed using Psycho‐Physiological Modeling (PsPM 6.0.0 available at pspm.sourceforge.net; Bach et al. 2018) in Matlab 2022b (Mathworks, Natick, Massachusetts, USA). We employed a single‐trial GLM (Bach et al. 2009, 2013) comprising one regressor for each CS onset, one regressor for each US onset, one regressor for each startle probe, and a canonical skin conductance response function with time‐derivative (Bach et al. 2010) and a fixed response latency. Note that the inversion algorithm is not informed about CS type or presence of the US. Skin conductance response estimates evoked by CS onsets were range corrected for each participant according to the individual minimum and maximum response across stimuli (i.e., CS+, CS−) and the three experimental phases (i.e., SCR_i_ − SCR_min_/SCR_max_ − SCR_min_; Lykken and Venables 1971).

#### 2.2.3 Fear‐Potentiated Startle Responses (FPS)

In order to elicit eye blink startle responses a loud noise (40 ms; 104 dB) was presented binaurally through headphones (startle probe). We recorded electromyographic activity using 7 mm Ag/AgCl electrodes positioned ±1 cm below the pupil and 1 cm below the lateral canthus, the outer corner of the eye (Blumenthal et al. 2005). EMG signal was recorded and amplified using in‐house software (VSRRP). The output signal was digitized at 1000 Hz. The data was then pre‐processed and analyzed using PsPM (PsPM 6.6.0; Bach et al. 2018). The data was band‐pass filtered (cut‐off: 50 and 470 Hz, 4th order Butterworth filter) and a notch filter was applied to remove 50 Hz harmonics. The signal was rectified and smoothed using a low‐pass filter (cut‐off: 53.05 Hz, 4th order Butterworth filter; Khemka et al. 2017). Subsequently, the data was down sampled to 500 Hz. We estimated trial‐by‐trial startle responses by employing a single‐trial GLM with regressors for each startle probe onset, and a flexible response onset latency in a 100 ms time window after startle probe onset. The inversion algorithm is not informed about the trial type or presence of the US. The resulting startle response estimates were range‐corrected within each participant across stimuli (CS+, CS−, NA) and across the three experimental phases (i.e., FPS_i_ − FPS_min_/FPS_max_ − FPS_min_).

### 2.3 Design and Experimental Tasks

**FIGURE 1:**
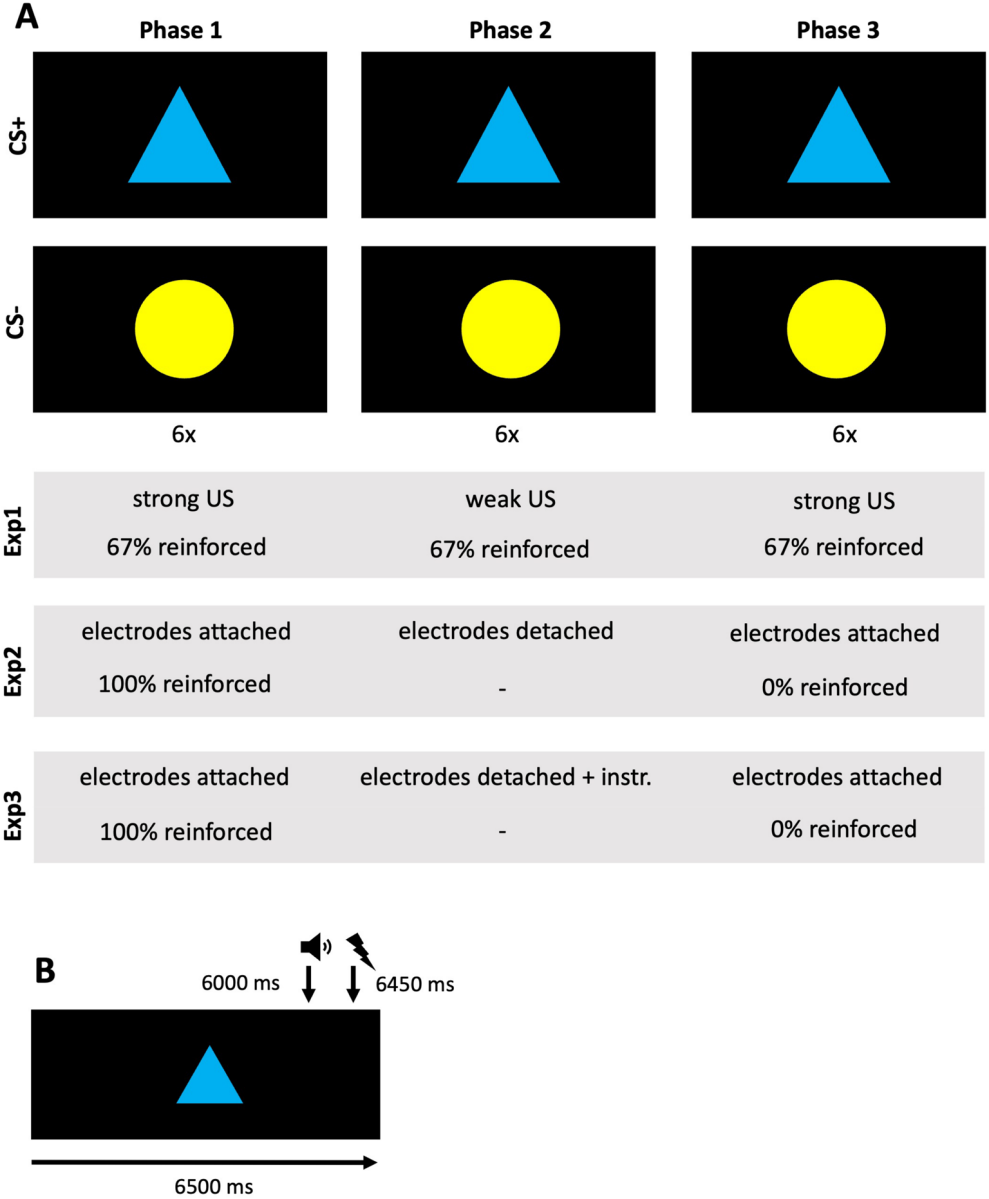
(A) Experimental design of all three experiments with differences between experiments in reinforcement and US devaluation manipulation pointed out in the gray boxes. The reinforcement of the CS+ varied per experiment whereas the CS− was never reinforced. (B) Trial timings for a reinforced trial. Timing for unreinforced trials was the same, except no US was presented.

Data for the current experiments were collected between March and June 2019. We conducted three within‐subjects differential fear‐conditioning studies consisting of three phases: a conditioning phase, a US devaluation phase, and a revaluation phase (see Figure 1). Each phase comprised six CS+ trials, six CS− trials, and 6 presentations of the startle probe alone (noise alone; NA). Conditioned stimuli were presented on the screen for 6500 ms. The startle probe was presented at 6000 ms and the US at 6450 ms, if presented. Inter‐trial intervals (ITI) had a randomized duration of 15–18 s. Before the start of each task, 10 startle probes were presented to reduce habituation. Trial order was randomized in such a way that not more than two trials of one type were presented after each other. Details that are specific to the experiment are discussed below.

#### 2.3.1 Experiment 1—Instructed US Devaluation

The devaluation manipulation in Experiment 1 was instructed, meaning that participants were informed and would experience that the US would be presented at the weak level (see *Stimuli*). Participants were instructed that one of the figures they would see on the screen could sometimes be followed by a strong or weak electric stimulus whereas the other figure would never be followed by an electric stimulus. Participants were also informed that the experiment consisted of three phases and that the intensity of the US could be either strong or weak in a phase, but that they would always be instructed about the intensity of the US before the start of each phase. In each phase, 4 out of 6 CS+ trials (66%) were reinforced in random order. Before the start of each phase, participants were informed through written instructions on screen that US intensity would be either strong or weak in the subsequent phase (strong in phase 1 and 3, weak in phase 2).

#### 2.3.2 Experiment 2—Actual US Devaluation

In contrast to Experiment 1, where we manipulated the expected value of the US through instructions, US devaluation in Experiment 2 consisted of removing the US electrodes without instructing participants. This should eliminate participants' expectation that the US will occur, thereby reducing the expected value via expectancy rather than through changing the US's intrinsic aversiveness. Before starting the task, participants were told that one figure could be followed by the electrical stimulus whereas the other figure would never be followed by the stimulus. To maximize expectations about the US occurrence, the CS+ was 100% reinforced in the first phase and not reinforced in the third phase. After the first phase ended, participants received written instructions on the screen that the experimenter would enter the room and were asked to remain seated. The experimenter then removed the electrodes without communication with the participants. The participants were instructed on the screen that the experiment would continue. At the end of the last phase the electrodes were re‐attached in a similar fashion. No more USs were presented in the final phase of the experiment.

#### 2.3.3 Experiment 3—Actual US Devaluation With Instructions

Although participants reported high confidence that they could no longer receive electrical stimuli when the electrodes were detached, in a pilot study including US expectancy ratings we experienced that many participants somehow still expected to receive an electrical stimulus after the electrodes were detached. For this reason, we replicated Exp2 exactly with the one exception that all participants were now instructed on screen at the start of phase two that the electrodes were detached and that there was no longer a risk of receiving an electrical stimulus. At the start of phase three, the participants were instructed that the electrodes were re‐attached and that the experiment would continue.

### 2.4 Procedure

The procedure was the same for all three experiments. All participants came to the lab for one session, were informed about the study and signed informed consent. To be able to exploratively investigate individual differences in learning based on character traits, all participants completed the state and trait anxiety inventory (STAI; Spielberger 1983) and anxiety sensitivity index (ASI; Peterson and Reiss 1992). Afterwards, all electrodes were attached, and the electrical stimulus was calibrated following the procedure outlined above. Participants then received instructions (see design for relevant instructions) and started the experimental task. After completing the task, participants completed ratings about the different CSs and their beliefs about the task.

### 2.5 Statistical Analyses

We first performed classical hypothesis testing on our data, investigating the immediate effect of US devaluation and revaluation on FPS responses and SCRs. Given that all experiments were similar in design except for the US devaluation manipulation, the data for all three experiments were analyzed in the same manner, separately for FPS and SCR responses. Data were analyzed using multilevel models with trial and stimulus as fixed within‐subject factors, nested within subjects. We tested whether acquisition was successful in phase 1 through a Trial (1–6) × Stimulus (CS+, CS−) interaction, where trial was included as a continuous predictor. To test the effect of US devaluation on the physiological responses, we compared the average conditioned response on the last three trials of phase 1 with the first trial of phase 2 by adding Stimulus (CS+, CS−) and Phase (late phase 1, early phase 2) as factors to the model. We expected that SCRs would show an instant reduction in differential responses while FPS responses would remain intact. We tested the effect of US re‐evaluation on the conditioned responses in the third phase through a Stimulus (CS+, CS−) × Phase (last three trials of phase 2, first trial of phase 3) interaction, and expected to observe an immediate return of fear for SCRs, and not for FPS responses. We averaged the last three trials of each phase to best capture what has been learned in that phase. Given the partial reinforcement schedule in Experiment 1, this approach provides a more stable estimate of conditioning, minimizing variations between participants due to different reinforcement schedules (two out of the last three trials of each phase were reinforced, but the exact schedule was random). We analyzed only the first trial at the start of a new phase, as this index served as a test of the US devaluation or revaluation manipulation.^1^ By focusing on the first trial, we avoided potential confounding effects of rapid model‐free learning. To be consistent, we continued this approach in Experiment 2 and 3, even though a full reinforcement schedule was employed. Where needed to further understand the data, we performed simple effect analyses or visual inspection of the data when interactions were significant. All analyses were conducted in RStudio (R version 4.5.1) using the *rstatix, lme4*, and *lmerTest* packages (Bates et al. 2015; Kassambara 2023; Kuznetsova et al. 2017).

#### 2.5.1 Computational Modeling

In addition to conventional statistical analyses, FPS and SCR data were analyzed using a hybrid computational model that combined model‐free and model‐based learning components. The model included a weighting parameter (*ω*) that indexed the relative contribution of model‐based versus model‐free control (e.g., Daw et al. 2011; Gläscher et al. 2010), with values ranging from 0 (purely model‐free) to 1 (purely model‐based). The model was fitted separately to each participant's FPS and SCR data using maximum‐likelihood estimation. Parameters were assumed to remain constant across the experiment. To assess differences between physiological measures, we compared *ω* estimates in a generalized linear mixed model using the *glmmTBM* package (Brooks et al. 2026), with measure (FPS versus SCR) added as fixed effect, and a random intercept per participant. Given that *ω* estimates are bounded between 0 and 1 and include boundary values, we fitted an ordered beta distribution. We expected to observe lower *ω* estimates (i.e., more model‐free control) for FPS responses compared to SCRs. To evaluate whether the hybrid model was indeed a good fit for the data, we compared its fit to a linear model that captured trial‐wise changes in physiological responses, but did not distinguish between CS+ and CS− trials, essentially capturing habituation. Full details of the model specification and fitting procedure are provided in the Supporting Information.

## 3 Results

### 3.1 Demographics and Ratings

Demographics and stimulus ratings of participants in all three experiments can be found in Table 1. Demographics and ratings were missing for two participants in Experiment 1 and one participant in Experiment 2. Noticeably, participants' anxiety sensitivity scores (ASI) were significantly higher in Experiment 3,^2^ and US intensity and ratings were significantly lower in Experiment 1. In both Experiment 2 and Experiment 3, we asked participants post hoc about their expectations during the US devaluation and re‐evaluation phases, but the questions were phrased slightly differently for each experiment. Participants in Experiment 2 reported overall high confidence (range 0–10) of not being able to receive an electric shock after electrode detachment (*M* = 8.6, SD = 2.5) and high confidence of being able to receive an electric shock again after re‐attachment (*M* = 7.9, SD = 2.1). In Experiment 3, participants reported a mean post hoc expectation (range 0–10) of receiving an electric shock after electrode detachment of 2.1 (SD = 2.4). We further asked for the participants' expectation of receiving an electrical stimulus at the start of the re‐evaluation phase for each CS separately to assess generalization. Mean US expectancy ratings were 6.8 (SD = 2.6) for the CS+ and 5.1 (SD = 3.0) for the CS−.

### 3.2 Experiment 1—Instructed US Devaluation

**FIGURE 2:**
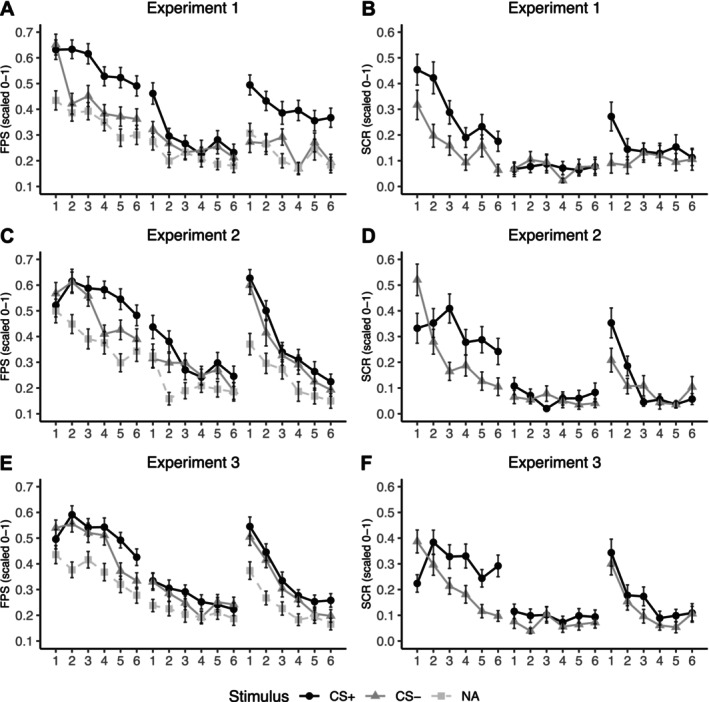
Fear‐potentiated startle (FPS; 2A, 2C, 2E) and skin conductance responses (SCR; 2B, 2D, 2F) for all three experiments plotted over time (three phases of six trials). All data are range‐corrected from 0 to 1. Error bars represent standard errors of the mean (SEM).

Multilevel models including Stimulus, Trial, and their interaction did not show a significant Stimulus × Trial interaction for either FPS (*β* = 0.01, *t*(398.0) = 1.34, *p* = 0.180) or SCR responses (*β* = −0.02, *t*(398.0) = 1.27, *p* = 0.205) in Phase 1. However, in both models, effects of Stimulus were significant, indicating that participants showed stronger FPS (*β* = 0.13, *t*(398.0) = 6.61, *p* < 0.001) and SCR (*β* = 0.12, *t*(398.0) = 5.52, *p* < 0.001) responses to the CS+ than the CS− (see Figure 2A,B). As hypothesized, instructing participants about the reduction of US intensity instantly reduced differential SCRs at the start of Phase 2 compared to late Phase 1 (Stimulus × Phase: *β* = −0.09, *t*(78.0) = 2.19, *p* = 0.031). Indeed, on the first Phase 2 trial, SCRs no longer differed between CS+ and CS− (*t*(78) = 0.06, *p* = 0.955, *d* = 0.01). In contrast, differential FPS did not reduce as a result of the instruction (Stimulus × Phase: *β* = −0.00, *t*(117.0) = 0.05, *p* = 0.959). In line with our predictions, there was still a significant CS+ > CS− difference on the first Phase 2 trial (*t*(78) = 3.77, *p* < 0.001, *d* = 0.84). In contrast to our predictions, the instruction that US intensity would increase in the third phase affected both differential SCR (Stimulus × Phase: *β* = 0.17, *t*(117.0) = 3.11, *p* = 0.002) and FPS (Stimulus × Phase: *β* = 0.21, *t*(117.0) = 3.87, *p* < 0.001), in line with model‐based patterns. In sum, the predictions for US devaluation were confirmed by the data, showing that while SCRs reduced instantly upon instructed US devaluation, differential FPS responses only reduced after experiencing the lower US intensity. Yet in the re‐evaluation phase, differential conditioned responses instantly recovered for both SCRs and FPS responses.

**FIGURE 3:**
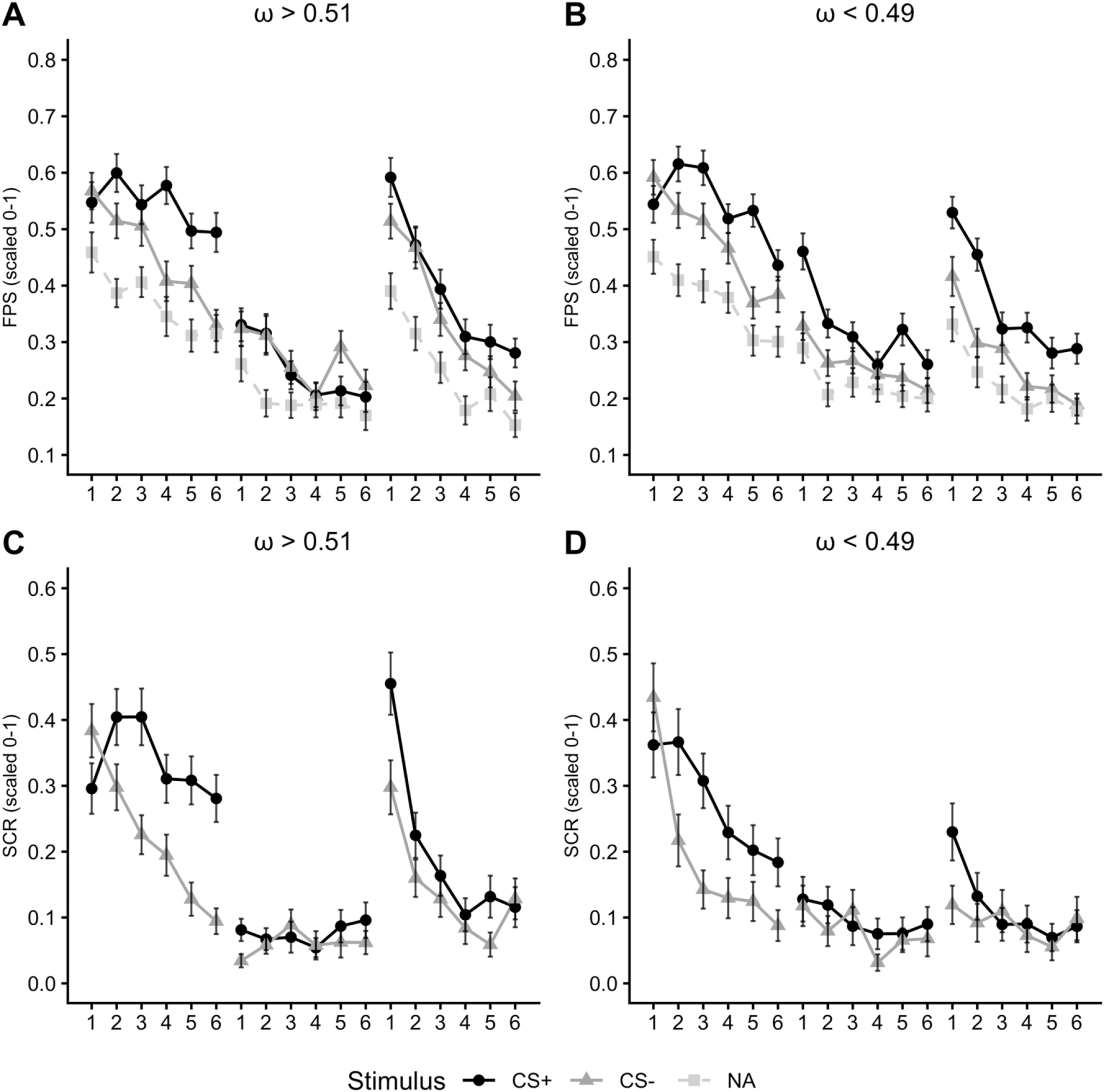
Grouping participants as more model‐free or more model‐based shows two distinctive profiles for FPS data in the US devaluation phase (A, B) but not for SCR data (C, D).

### 3.3 Experiment 2—Actual US Devaluation

In the second experiment we investigated whether US electrode removal before Phase 2 as a means of actual US devaluation would also differentially affect SCRs versus FPS responses. Fear acquisition was successful for both SCR (Stimulus × Trial: *β* = 0.05, *t*(388.0) = 3.28, *p* = 0.001) and FPS (Stimulus × Trial: *β* = 0.03, *t*(388.0) = 2.78, *p* = 0.005; Figure 2C,D) in Phase 1. Next, we tested whether the CS+ > CS− difference would be eliminated after US devaluation (model‐based) or continue to exist (model‐free). In contrast to our expectation, the Stimulus × Phase interaction was not significant (*β* = −0.09, *t*(76.0) = 1.83, *p* = 0.070) for SCRs, although simple effects did show that differential responding was significant at the end of Phase 1 (*t*(76) = 3.87, *p* < 0.001, *d* = 0.88), but not on the first trial of Phase 2 (*t*(76) = 1.27, *p* = 0.208, *d* = 0.29), showing some support for a model‐based pattern. For FPS responses, we found no evidence for an immediate reduction in FPS responses (Stimulus × Phase: *β* = −0.01, *t*(76.0) = 0.10, *p* = 0.918), and still observed a significant CS+ > CS− difference on the first Phase 2 trial (*t*(76) = 3.14, *p* = 0.002, *d* = 0.71). As predicted, the re‐attachment of the US electrodes in phase three resulted in a significant differential increase in SCRs (Stimulus × Phase: *β* = 0.12, *t*(114.0) = 2.52, *p* = 0.013). Interestingly, electrode reattachment also resulted in a strong but stimulus‐unspecific increase in FPS responses (Stimulus × Phase: *β* = 0.00, *t*(76.0) = 0.04, *p* = 0.966; Phase 3: *β* = 0.36, *t*(73.0) = 9.48, *p* < 0.001). In sum, our data largely support the model predictions for US devaluation, with SCRs reflecting model‐based learning and FPS responses reflecting model‐free learning. In contrast, reattachment of the electrode resulted in an immediate return of differential SCRs, in line with model‐based learning, and a stimulus‐unspecific increase in FPS responses.

### 3.4 Experiment 3—Actual US Devaluation With Instructions

In the final experiment we added instructions to the US devaluation procedure to enhance the confidence that participants would have in not receiving an electrical stimulus in phase two. As shown in Figure 2E,F, overall fear acquisition was successful for SCRs (Stimulus × Trial: *β* = 0.06, *t*(518.0) = 4.88, *p* < 0.001) and FPS responses (Stimulus × Trial: *β* = 0.03, *t*(569.0) = 2.50, *p* = 0.013). In line with our expectations, SCRs showed an immediate reduction of differential responding following instructed US electrode removal (Stimulus × Phase: *β* = −0.12, *t*(102.0) = 2.58, *p* = 0.011) and there was no longer a CS+ > CS− difference on the first Phase 2 trial (*t*(102) = 1.23, *p* = 0.223, *d* = 0.24). For FPS responses, there was no significant Stimulus × Phase interaction (*β* = −0.08, *t*(143.9) = 1.54, *p* = 0.126). Yet in contrast with model‐free learning, simple effects showed that while differential responding was significant at the end of Phase 1 (*t*(102) = 2.29, *p* = 0.024, *d* = 0.45), it was not on the first Phase 2 trial (*t*(102) = 0.11, *p* = 0.911, *d* = 0.02). However, the CS+ responses were still higher than the noise alone trials on the first trial (*t*(204) = 2.98, *p* = 0.009, *d* = 0.58). The reattachment of the US electrode before the third phase resulted in a strong, but stimulus‐unspecific increase for both SCRs (Stimulus × Phase: *β* = 0.02, *t*(153.0) = 0.41, *p* = 0.682; Phase 3: *β* = 0.24, *t*(78.6) = 4.59, *p* < 0.001) and FPS responses (stimulus × phase: *β* = 0.03, *t*(153.0) = 0.65, *p* = 0.514; Phase 3: *β* = 0.28, *t*(120.7) = 7.62, *p* < 0.001).

### 3.5 Computational Modeling of Data

Across experiments (*n* = 131) *ω*‐estimates for SCRs (*M* = 0.52, SD = 0.34) and FPS responses (*M* = 0.47, SD = 0.31) did not significantly differ (*b* = 0.24, *z* = 1.77, *p* = 0.077). Due to a rounding error in the model fitting, one *ω*‐estimate was < 0 (−2.06e−18) and was replaced with 0. Interestingly, exploratively categorizing participants as model‐free (*ω* < 0.49) versus model‐based (*ω* > 0.51) for each measure demonstrated different visual profiles (see Figure 3). For FPS data the patterns clearly differed and matched either model‐based (immediate drop in FPS response at the start of Phase 2; *n* = 56; Figure 3A) or model‐free learning (no reductions of responding after US devaluation; *n* = 72; Figure 3B). For SCRs, participants who were categorized as model‐based learners (*n* = 64; Figure 3C) exhibited a clear pattern in line with model‐based learning. Yet responses of participants classified as model‐free learners (*n* = 56; Figure 3D) did not follow a model‐free pattern in Phase 2, but rather an overall reduction in conditioned responding throughout the experiment. A robustness check showed that these patterns were not sensitive to the specific cut‐off value.

#### 3.5.1 Model Selection Uncertainty

When comparing summed negative log likelihood (nLL) values between the hybrid model and a simple linear model, the hybrid MF‐MB model provided a better fit than the linear model for both SCR data (nLL_hybrid_ = −611.8; nLL_linear_ = −357.1) and FPS data (nLL_hybrid_ = −844.3; nLL_linear_ = −542.7). However, taking model complexity into account, the simpler linear model in fact outperformed the hybrid model for both SCRs (AIC_hybrid_ = 348.4; AIC_linear_ = 71.7) and FPS responses (AIC_hybrid_ = −116.5; AIC_linear_ = −299.4). These results indicate that even though the hybrid model provides a better fit to the data, this does not outweigh its greater complexity. A simpler learning‐independent model may thus provide a more parsimonious account of the data.

### 3.6 Exploration of Individual Differences

Given the apparent individual differences in the extent to which FPS responses reflected model‐free versus model‐based learning, we explored whether omega estimates were associated with individual characteristics in three ordered beta mixed models including one measure (FPS vs. SCR) × anxious trait (trait anxiety, state anxiety, or anxiety sensitivity) interaction per model. Anxious traits were standardized before being added to the model. Because impairments in model‐based control have been proposed as a core feature of psychopathology (Voon et al. 2017), higher levels of anxious traits may be related to more model‐free learning. However, *ω* estimates were not significantly predicted by trait anxiety, state anxiety, or anxiety sensitivity, nor were there any interactions with measure (FPS or SCR; all *p*s > 0.213; see Supporting Information).

## 4 Discussion

To gain a more detailed understanding of model‐free and model‐based fear learning processes in human fear‐conditioning, we evaluated the effect of (instructed) US devaluation on FPS responses and SCRs in three experiments. All experiments included a conditioning phase, a devaluation phase, and a revaluation phase. US devaluation occurred either through instructions (Exp1), the detachment of the US electrode (Exp2), or a combination of both (Exp3). In line with our expectations, we found that SCRs instantly reduce to baseline (CS−) levels of responding after US devaluation in Experiment 1 and 3, reflecting model‐based learning. In the second experiment, however, there was no clear evidence for a model‐based pattern in the SCR data. FPS responses remained elevated at the start of the devaluation phase in Experiment 1 and 2, in line with model‐free learning. In the third experiment, where US electrode removal was combined with instructions, FPS responses also decreased instantly, suggesting model‐based control. For US revaluation (increase in US intensity or re‐attachment of the US electrode), both SCR and FPS patterns were more reflective of model‐based control, showing immediate differential or non‐differential increases in conditioned responding at the start of the third phase.

The observation that SCRs instantly decrease upon devaluation of the US in the first and third experiment is in support of existing literature (Costa et al. 2015; Hosoba et al. 2001; Javanbakht et al. 2017; Luck and Lipp 2015a, 2015b; Mertens et al. 2016; Schultz et al. 2013; Sevenster et al. 2012). These findings strongly suggest that SCRs reflect model‐based learning during Pavlovian conditioning, in line with previous results showing that SCRs during fear conditioning and extinction are best fit by a model‐based model (Letkiewicz et al. 2022). In the second experiment, the model‐based pattern in the SCR data was weaker, as the expected interaction was not significant. We did, however, see a reduction of SCRs at the start of the devaluation phase, as evidenced by the absence of a significant difference between the CS+ and CS− on the first trial, supportive of a model‐based account. In contrast, FPS responses appear to reflect a mixture of model‐based and model‐free learning. In the first two experiments, FPS responses were larger to the CS+ than to the CS− at the start of the devaluation phase, following model‐free learning. Yet in Experiment 3, FPS responses showed immediate reductions upon US electrode removal, although not to the level of the startle probe alone (i.e., full baseline), reflecting more model‐based learning. The observation that FPS responses can reflect both model‐free and model‐based learning is in support of previous studies that demonstrated both delayed and immediate updating of FPS responses based on instructions (Costa et al. 2015; Mertens and De Houwer 2016; Sevenster et al. 2012). In sum, while SCRs seem best explained by model‐based learning, FPS responses may reflect a mixture of model‐free and model‐based control.

A potential factor that could explain why FPS responses immediately reduced at the start of the devaluation phase in the third experiment is uncertainty. Conditioned responses in aversive learning reflect an interaction between outcome probability and experienced intensity, rather than probability alone. While SCRs appear particularly sensitive to changes in US probability, FPS responses seem to capture the acquired reflexive affective value of the US (Hamm and Weike 2005). Critically, in the case of a very small but non‐zero probability of an aversive outcome, this affective value may dominate the FPS response. On the other hand, under conditions of near‐absolute certainty that the US will not occur, little affective value remains to sustain FPS responding. The sole difference between Experiment 2 and 3 was that US electrode removal was accompanied by explicit instructions that the US would no longer occur, which may have increased participants' confidence in the manipulation and allowed model‐based updating to emerge more clearly. While in Experiment 2 one could argue that US electrode removal resulted in high certainty of no longer receiving the US, a post‐experiment interview indicated that many participants still had some expectations that a US would occur at the start of the devaluation phase, for example through the SCR electrodes. In sum, while not explicitly manipulated or measured, it could be that uncertainty or trust in the manipulation determines the balance between model‐free and model‐based control of FPS responses.

Another interesting observation is that US revaluation (i.e., instructions that the strong US will be presented again or reattachment of the US electrode) results in an immediate increase of both FPS responses and SCRs. Based on a model‐free framework, one would expect to observe an increase in conditioned responding only after experiencing the CS‐US reinforcement again (and thus not at all in Experiment 2 and 3). Upon US revaluation, FPS responses do thus not reflect model‐free learning. This finding is also in line with previous work showing the FPS resposnes increase upon instructed CS‐US contingencies (Costa et al. 2015). Interestingly, a similar pattern is observed in maladaptive behaviors commonly seen in clinical settings, which often prove resistant to change even when the absence of threat is experienced, and are prone to re‐emerge without the re‐experiencing of the original threat. Pr Such behaviors cannot be accounted for by a purely model‐free framework, yet do fit with the idea that the fear‐potentiated startle is a defensive response (Hamm 2015). From an evolutionary perspective, defensive responses may increase upon even the smallest possibility that a threat may occur. Importantly, these results also show that fear learning and unlearning may be governed by different learning processes. An unexpected but interesting finding is that FPS responses and SCRs to the CS− also increased upon US revaluation in Experiment 2 and 3. This is likely explained by the fact that the continued presentation of the weak US in the devaluation phase in Experiment 1 resulted in additional safety learning for the CS−. The absence of continued safety learning in Experiment 2 and 3 may have increased the ambiguity of the CS− at the start of the US devaluation phase, resulting in elevated US responses. It should be noted, however, that Experiment 1 also employed a different reinforcement schedule during acquisition, making it difficult to attribute differences between experiments solely to the US devaluation phase. Another possible explanation is the fixed order of the devaluation and revaluation phase in the experiments. While participants may have experienced some uncertainty or distrust regarding US devaluation (phase 2), their confidence may have been higher in the revaluation manipulation as it was always presented second (phase 3), resulting in an immediate increase in conditioned responding. Future studies could implement multiple US devaluation and revaluation phases to further isolate model‐free learning from trust or uncertainty.

In addition to group‐based analyses, we also fitted a hybrid model‐free/model‐based model to each participant's individual data (Daw et al. 2011). This model included a parameter (*ω*) determining the balance between model‐free and model‐based control. Surprisingly, we found no significant differences between *ω*‐estimates for FPS responses and SCRs, suggesting minimal differences between model‐free and model‐based control. Yet splitting participants into two groups based on their *ω*‐estimates demonstrated two distinct visual profiles for FPS data, but not for SCRs. Although explorative, this suggests considerable individual variability in the extent to which FPS responses reflect model‐free versus model‐based learning. Interestingly, in contrast with prior evidence linking impairments in model‐based control to psychopathology, this variability was not related to individual differences in anxiety‐related traits (Cisler et al. 2024; Voon et al. 2017). These analyses, however, were potentially underpowered, as larger sample sizes and more variety in anxious traits may be necessary to provide robust estimates (Schönbrodt and Perugini 2013). What further complicates the interpretation of these modeling results is that model‐free and model‐based learning only dissociate on trials immediately after US devaluation or revaluation, and patterns look similar during acquisition and towards the end of each phase. While computational modeling of the data can provide valuable insights into underlying processes, differences across the full response trajectory may appear minimal, as only a small number of trials are expected to show divergent responses. Furthermore, it should be noted that although the hybrid model provided a better absolute fit for the data, this did not outweigh its additional parameters, and a simple linear model provided a more parsimonious account of the data. This result may, however, also reflect differences in how the models treat time: the hybrid model did not incorporate habituation, whereas the linear model did.

These results should be interpreted in light of some limitations. First, we did not assess whether participants believed the instructions in Experiment 1, even though the manipulation was entirely based on instructions. In Experiment 2 and 3, expectancy or confidence ratings were measured post hoc, potentially introducing bias. Future studies could benefit from incorporating more reliable US expectancy or trust ratings to further elucidate the role of uncertainty in the balance between model‐based and model‐free responses. Second, the three experiments employed different reinforcement schedules. Where we used partial reinforcement throughout Experiment 1 to avoid reductions in US value due to habituation, we changed the acquisition reinforcement rate to 100% in Experiment 2 and 3 to enhance the effect of US devaluation by removing the electrode. While these were deliberate design choices intended to strengthen the manipulation in each experiment, they complicate cross‐experiment comparisons and limit the extent to which diverging results can be attributed specifically to the US devaluation manipulation.

A more general, but important limitation, is that physiological measurements are inherently noisy, which may compromise their interpretability (Ney et al. 2018). We aimed to address this by using GLM‐based inference of single‐trial responses from continuous physiological data (Bach et al. 2009, 2013). Variability in the signal due to external factors (e.g., room temperature) or internal processes (e.g., attention), however, was not accounted for. It should therefore be noted that differences observed between FPS responses and SCRs may also be explained by psychometric properties of these measures rather than different learning systems. Moreover, observed differences could reflect the degree to which each measure captures distinct aspects of the learning process (e.g., arousal versus affective valence, or prediction versus surprise), rather than an underlying learning system (Ojala and Bach 2020). For example, Leuchs et al. (2019) found that FPS responses and SCRs correlated only weakly in a fear‐conditioning task that did not include a model‐based manipulation, suggesting that other factors could also drive the dissociation between these measures. Further clarifying which learning systems and learning properties are reflected by FPS responses and SCRs is therefore an important direction for future research. Even when the exact mechanisms remain unclear, our results do indicate that SCRs are more sensitive to instructions or other non‐experiential manipulations than FPS responses, which has important implications for the interpretation of fear‐conditioning results.

Understanding the contribution of model‐free and model‐based learning to fear‐conditioning is especially relevant when considering the modification of already learned behaviors, as is often the case in clinical practise. Given that most therapies—and exposure treatment in particular—make use of experiential learning, it is likely that model‐free learning contributes at least to some extent to therapeutic change. We thus suggest that clinically relevant insights into fear extinction should not be derived exclusively from SCR data, and highlight the translational relevance of FPS responses. On the other hand, when evaluating manipulations that rely on model‐based learning strategies (e.g., cognitive restructuring), one may want to include SCRs, as FPS responses could fail to capture these effects. Ultimately, these decisions depend on the specific research question and experimental manipulation. Lastly, we focused on FPS and SCR because they are widely used in fear conditioning and can dissociate under certain circumstances, but other measures may also index model‐free and/or model‐based learning. For example, pupil dilation decreases immediately when US electrodes are removed and may therefore reflect model‐based control (Ojala and Bach 2020; Visser et al. 2013). By contrast, CS liking may show a more model‐free profile, as it appears resistant to instructed extinction (Luck and Lipp 2015a). Clarifying which learning processes are captured by different physiological and behavioral indices will guide researchers in selecting appropriate outcome measures and interpret fear‐conditioning findings more precisely.

In conclusion, in support of existing literature, our findings suggest that SCRs primarily reflect model‐based learning during human fear‐conditioning, whereas FPS responses appear to capture both model‐free and model‐based learning patterns. It remains unclear, however, what experimental and individual characteristics determine the balance between model‐free and model‐based control over FPS responses. Moreover, there could be alternative learning mechanisms that drive either response system. That said, our data do suggest that FPS responses are more sensitive to capturing persistent fear responses, which are a core characteristic of anxiety disorders. Ultimately, distinguishing different types of associative learning and the measures that reflect them will guide researchers not just in selecting appropriate outcomes measures in experimental studies, but also help to design interventions that more specifically target the learning mechanisms underlying fear and anxiety disorders.

## Author Contributions

**L. E. Stemerding:** investigation, data curation, formal analysis, visualization, writing – original draft. **F. L. Reinhold:** investigation, data curation. **M. Kindt:** conceptualization, supervision, funding acquisition, writing – review and editing. **A. M. V. Gerlicher:** conceptualization, methodology, formal analysis, supervision.

## Funding

This work was funded by an ERC advanced grant (74326) awarded to M. Kindt.

## Conflicts of Interest

The authors declare no conflicts of interest.

## Supporting information


**Figure S1:** Simulations of model‐free (*ω* = 0) and model‐based (*ω* = 1) learning using the task structures of Experiment 1 (A, B) and Experiments 2 and 3 (B, C). Experiment 2 and 3 shared an identical task design, and only differed in terms of additional instructions in Experiment 3, which are not included in the model. The *ω*‐parameter was fixed at 0 in the model‐free simulations, reflecting exclusive model‐free control, and fixed at 1 in the model‐based simulations, reflecting exclusive model‐based control. For each task structure and *ω*‐parameter, we generated 100 simulated datasets using the trial sequence and reinforcement rates of each task structure. The model‐free learning rate (*α*) and the model‐based learning rate (*η*) were independently sampled for each simulation from a uniform distribution [0,1]. Plot lines represent the average of the 100 simulations; shaded areas represent standard errors of the mean.
**Figure S2:** Distribution of omega estimates across experiments (*n* = 131) for both SCR and FPS data.
**Table S1:** Main and interaction effects from the ordered beta mixed models testing the relationship between FPS and SCR omega estimates and individual characteristics.
**Table S2:** Trial level contrasts (CS+ vs CS‐) for single trial comparisons from a full multilevel model.

## Data Availability

The data that support the findings of this study are openly available in OSF at https://osf.io/tw3rb/.

## Endnotes

^1^
Given that physiological data at the single‐trial level can be noisy, we conducted robustness checks by analyzing all trials using a multilevel model. The trial‐level contrasts obtained from this model were consistent with the single‐trial comparisons. The results can be found in the Supporting Information.

^2^
To control for potential effects of ASI, we performed sensitivity analyses in which ASI was included as a covariate in the multilevel models. This did not change any of the results.
