# Supplementary material for: Model‐Free and Model‐Based Learning in Human Fear Conditioning
Source: Psychophysiology. 2026 Jun 30;63(7):e70349. doi: 10.1111/psyp.70349 (PMC13317758; doi:10.1111/psyp.70349)
Supplement: Supplementary file 1 — Figure S1: Simulations of model‐free (ω = 0) and model‐based (ω = 1) learning using the task structures of Experiment 1 (A, B) and Experiments 2 and 3 (B, C). Experiment 2 and 3 shared an identical task design, and only differed in terms of additional instructions in Experiment 3, which are not included in the model. The ω‐parameter was fixed at 0 in the model‐free simulations, reflecting exclusive model‐free control, and fixed at 1 in the model‐based simulations, reflecting exclusive model‐based control. For each task structure and ω‐parameter, we generated 100 simulated datasets using the trial sequence and reinforcement rates of each task structure. The model‐free learning rate (α) and the model‐based learning rate (η) were independently sampled for each simulation from a uniform distribution [0,1]. Plot lines represent the average of the 100 simulations; shaded areas represent standard errors of the mean. Figure S2: Distribution of omega estimates across experiments (n = 131) for both SCR and FPS data. Table S1: Main and interaction effects from the ordered beta mixed models testing the relationship between FPS and SCR omega estimates and individual characteristics. Table S2: Trial level contrasts (CS+ vs CS‐) for single trial comparisons from a full multilevel model. [file PSYP-63-e70349-s001.docx]

**Model-free and model-based learning in human fear conditioning**

Stemerding LE, Gerlicher AMV, Reinhold FL, Kindt M

Supplementary Materials

06.2026

**Computational modelling**

To understand learning processes driving physiological responses, we modelled the participant level FPS and SCR data to a hybrid model-free/model-based model including a weighing parameter (omega). Model implementation and parameter fitting was conducted in R using R Studio (version 2024.09.0). The model was optimized by minimizing the negative log likelihood (nLL) between the model prediction for each trial and the trial-level standardized physiological responses using the R function *optim*. To avoid local minima in parameter fitting, we initiated optimization with 50 different start values of the parameter. Model-free learning was formalized using a Rescorla-Wagner learning rule (Rescorla & Wagner, 1972) and model-based learning was formalized using transition-probabilities model adapted from Schad et al., (2019). See model specifications below. Associative strength (V) on each trial was defined as:

$$V_{t}=\omega*V_{t,mb}+\left( 1-\omega\right)*V_{t,mf}$$

*Model-free Rescorla-Wagner model*

The Rescorla and Wagner model postulates that the associative strength of the CS is updated on each trial by the prediction error multiplied by a learning rate, where the prediction error term is defined as the outcome minus the associative strength of the current trial:

$$V_{t+1}=\alpha*(R- V_{t})$$

Where V is the associative strength, R the outcome (1 when the US is presented and 0 when the US is not presented). The learning rate ($\alpha$) is an individual parameter that is estimated in the model fitting process. For simplicity, we assumed that the learning rate is the stable throughout the learning process for both CSs. We further assumed that participants had no specific expectations about the two stimuli and initialized both CSs and the electrode to a start value of 0.5.

*Model-based transition probabilities model*

In its basis, the transition probabilities model is also governed by error-based learning, similar to a Rescorla Wagner learning rule, with the exception that environmental changes can be incorporated in the structure of the model. In this model, the associative value of the CS is determined by the probability that the CS is followed by a given outcome US/no US (based on a transition probabilities matrix) multiplied by a reward matrix, where reward is defined at 0 (no US) 1 (US):

$$V_{cs}= T_{cs}*Rmat$$

At each trial, the transition probability matrix is updated by the state prediction error multiplied by a learning rate:

$$T_{cs, t+1}=T_{cs,t}+ \eta*(1-T_{cs, t})$$

where T_cs,t_ is the estimated probability of the observed outcome at trial t, and $\eta$ is the estimated learning rate. The state prediction error (1 – T_cs,t_) represents the difference between the observed outcome and the expected probability of that outcome. At the given trial, the transition probability of the non-observed outcome (e.g., no US when the US was presented) was multiplied by 1-$\eta$. When a CS was not presented on a given trial (e.g., the CS- when the CS+ was presented), its transition probabilities were carried forward to the next trial. Critically, in Experiment, the reward value for the US was set at 0.01 at the start of the devaluation phase (trial 13), and back to 1 at the start of the third phase. In Experiment 2 and 3, the transition probabilities for both CSs were set to predict 'no shock' at the start of phase two. At the start of the third phase (trial 25), transition probabilities were restored to the values estimated at the end of the first phase (trial 12).

**Behavioural link function**

In line with previous work (Tzovara et al., 2018) we assumed that the physiological response of each trial is a linear function of the trial-specific value-estimate of the model-free or model-based learning model and an error term $\epsilon$:

$$y={\beta_{0}+ \beta}_{1}* V+ \epsilon$$

Where V is the modelled estimate of the value based on the given parameters and y the physiological response of the present trial. β_0_ and β_1_ are participant-specific free parameters. The negative log likelihood was calculated based on the fit of this final model.

**Simple linear model**

To test model selection uncertainty, we also fitted the data to a simple linear model, where t represents the trial number:

$$y={\beta_{0}+ \beta}_{1}* t+ \epsilon$$

We compared to model fit between this model and the hybrid model-free/model-based model using summed Akaike Information Criteria across participants.

**Model simulations**

Prior to fitting the model to empirical data, we conducted simulations to characterise the expected behaviour of the model under extreme values of ω (i.e., purely model-free or purely model-based control) given the structure of the task (Supplementary Figure 2). These simulations demonstrated that under purely model-based control (ω = 1), associative strength is immediately reduced to zero after US devaluation (Fig. 1B,D), whereas under purely model-free control (ω = 0), associative strength remains elevated during the initial trials of Phase 2 (Fig. 1A,C). During revaluation (phase 3), a model-free measure would show an increase of conditioned responding (B,D) whereas a model-free measure would remain at the level of late phase 2 for initial trials (A,C), and only increase upon re-experiencing the threat. In Exp2&3 (C), phase 3 is no longer reinforced, so conditioned responding should not return at all.

**Supplementary Figure 1.** Simulations of model-free (ω = 0) and model-based (ω = 1) learning using the task structures of Experiment 1 (A,B) and Experiments 2 and 3 (B,C). Experiment 2 and 3 shared an identical task design, and only differed in terms of additional instructions in Experiment 3, which are not included in the model. The ω-parameter was fixed at 0 in the model-free simulations, reflecting exclusive model-free control, and fixed at 1 in the model-based simulations, reflecting exclusive model-based control. For each task structure and ω-parameter, we generated 100 simulated datasets using the trial sequence and reinforcement rates of each task structure. The model-free learning rate (α) and the model-based learning rate (η) were independently sampled for each simulation from a uniform distribution [0,1]. Plot lines represent the average of the 100 simulations; shaded areas represent standard errors of the mean.

**
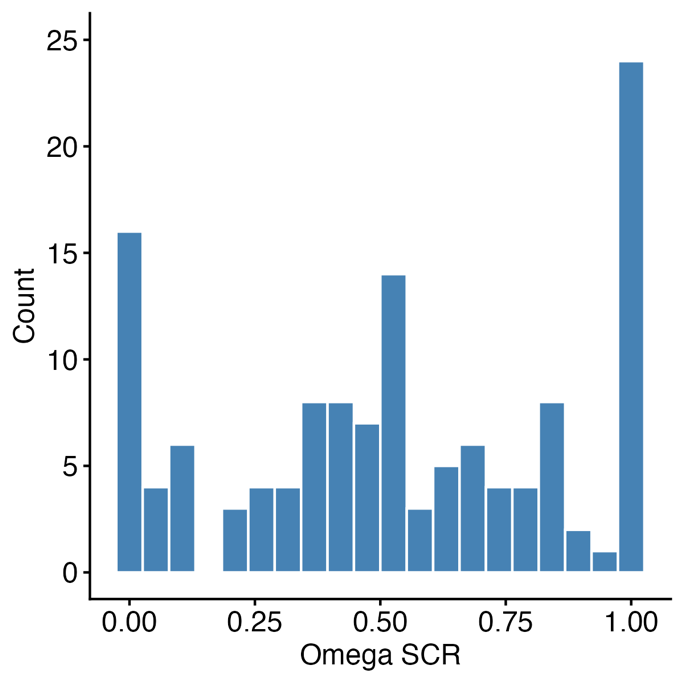

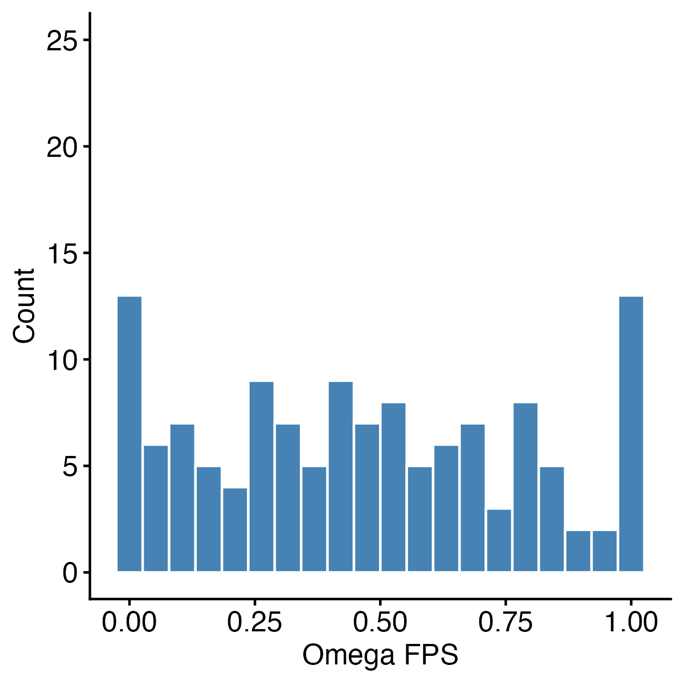
**

**Supplementary Figure 2.** Distribution of omega estimates across experiments (n = 131) for both SCR and FPS data.

**Individual differences**

Results of the ordered beta mixed models testing the effect of anxious traits on omega estimates for all participants (n = 131) are shown in Supplementary Table 1 below. Trait and state anxiety are measured with the State Trait Anxiety Index (STAI; Spielberger, 1983). Anxiety sensitivity is measured with the Anxiety Sensitivity Index (ASI; Peterson & Reiss, 1992). Higher omega-estimates indicate more model-based learning, one would thus theoretically expect a negative relationship between anxiety-related characteristics and omega estimates (i.e., more anxiety is related to more model-free learning). Because anxious traits tend to be strongly correlated, we fitted three separate ordered beta mixed models, one per anxious trait, including an interaction with Measure (FPS vs. SCR) and a random intercept per participant:

1. Omega ~ Measure (FPS, SCR) × Trait anxiety + (1 | subject)
2. Omega ~ Measure (FPS, SCR) × State anxiety + (1 | subject)
3. Omega ~ Measure (FPS, SCR) × Anxiety sensitivity + (1 | subject)

**Supplementary Table 1**

*Main and interaction effects from the ordered beta mixed models testing the relationship between FPS and SCR omega estimates and individual characteristics.*

|  | b | z | p-value |
| --- | --- | --- | --- |
| Trait anxiety | -0.13 | 1.05 | .293 |
| State anxiety | -0.15 | 1.15 | .252 |
| Anxiety sensitivity | 0.12 | 0.92 | .360 |
| Trait anxiety × Measure | 0.09 | 0.64 | .521 |
| State anxiety × Measure | 0.12 | 0.87 | .386 |
| Anxiety sensitivity × Measure | -0.17 | 1.25 | .213 |

**Trial-level contrasts**

Trial contrasts (CS+ vs CS-) from a full multilevel model fitted to all data points can be found in Supplementary Table 2.

**Supplementary Table 2**

|  | Estimate | t | p-value |
| --- | --- | --- | --- |
| **US devaluation** |  |  |  |
| Exp1 SCR | 0.007 | 0.20 | .846 |
| Exp1 FPS | -0.085 | 2.53 | **.011** |
| Exp2 SCR | -0.006 | 0.17 | .867 |
| Exp2 FPS | -0.076 | 2.23 | **.026** |
| Exp3 SCR | -0.038 | 1.19 | .234 |
| Exp3 FPS | -0.029 | 1.01 | .312 |
| **US revaluation** |  |  |  |
| Exp1 SCR | -0.117 | 3.17 | **.002** |
| Exp1 FPS | -0.186 | 5.51 | **<.001** |
| Exp2 SCR | -0.101 | 2.68 | **.007** |
| Exp2 FPS | -0.044 | 1.27 | .203 |
| Exp3 SCR | -0.050 | 1.56 | .120 |
| Exp3 FPS | -0.028 | 0.96 | .337 |

*Note.* US devaluation shows the CS+/CS- contrast on the first trial

of phase 1, US revaluation shows the first trial of phase 2.
